# The predictive value of sarcopenia and myosteatosis in trans-arterial (chemo)-embolization treated HCC patients

**DOI:** 10.18632/aging.205375

**Published:** 2024-01-05

**Authors:** Jing Long, Xin Zhang, Wei Mi, Jianjun Shi, Hongwei Ren, Qiang Wang

**Affiliations:** 1 Department of Interventional Radiology, Tianyou Hospital Affiliated to Wuhan University of Science and Technology, Hubei, P.R. China; 2 Department of Imaging, Tianyou Hospital Affiliated to Wuhan University of Science and Technology, Hubei, P.R. China

**Keywords:** sarcopenia, skeletal muscle index, psoas muscle index, myosteasis, prognosis, trans-arterial (chemo)-embolization, hepatocellular carcinoma

## Abstract

Background: We conducted a meta-analysis to provide evidence-based results for the predictive values of sarcopenia, skeletal muscle index, psoas muscle index and the myosteatosis regarding the impact of survival outcomes and tumor response in patients treated by trans-arterial (chemo)-embolization (TAE/TACE), thereby optimizing therapeutic strategies and maximizing clinical benefits for hepatocellular carcinoma patients.

Methods: Qualified studies were retrieved from PubMed, the Cochrane Library, EMBASE, and Google Scholar before June 19, 2023. We investigated the relationships between sarcopenia, SMI, PMI, myosteatosis, and the overall survival of TAE/TACE-treated hepatocellular carcinoma patients with pooling data.

Results: A total of 167 studies were collected and 12 studies were finally included for analysis. The meta-analysis assisted that the sarcopenia (HR: 1.46, 95% CI: 1.30–1.64, *p* < 0.001), skeletal muscle index (HR: 1.48, 95% CI: 1.29–1.69, *p* < 0.001), and psoas muscle index (HR: 1.45, 95% CI: 1.19–1.77, *p* < 0.001) were significantly related to a shorter OS of hepatocellular carcinoma patients who treated by TAE/TACE. Sarcopenia significantly contributed to a lower objective response rate of TAE/TACE treated hepatocellular carcinoma patients (OR: 0.80, 95% CI: 0.65–0.98, *p* = 0.032). But there was no significant association between the myosteatosis and the overall survival (HR: 1.29, 95% CI: 0.74–2.25, *p* = 0.366). Sensitivity analysis supported the stability and dependability of above analyses conclusions.

Conclusion: Sarcopenia, skeletal muscle index and psoas muscle index, are significant prognostic predictors for TAE/TACE treated hepatocellular carcinoma patients. While myosteasis does not demonstrate a prognostic impact on the overall survival of TAE/TACE treated hepatocellular carcinoma patients.

## INTRODUCTION

Liver cancer, of which hepatocellular carcinoma (HCC) is the most common type, is the second cause of cancer-related deaths worldwide in 2018 [1]. Even though transplant and surgical resection treatment are the most popular strategies for HCC, there are still considerable patients diagnosed at an advanced stage who have lost the chance [2]. In the past few years, trans-arterial (chemo)-embolization (TAE/TACE) has been a prioritized treatment option for HCC patients who were at an intermediate stage, and the strategy has been proven to be effective and safe [3]. Patients treated by TAE/TACE demonstrated an acceptable general status with a relatively lower rate of liver insufficiency [4].

Patients who were diagnosed with HCC usually suffered from changes in muscle, which included changes in quantity, such as sarcopenia, and changes in structural composition, such as myosteatosis, which could affect curative effectiveness of TAE/TACE in HCC patients. More than 90% of HCC patients were diagnosed with cirrhosis, which was usually associated with sarcopenia [5]. Sarcopenia was usually calculated by normalized cross-sectional muscle area at the level of L3 on an abdominal CT scan before embolization by patient’s height and was defined as a lower skeletal muscle index (SMI) or psoas muscle index (PMI) [6]. Recent studies pointed out that sarcopenia was related to a lower overall survival and objective response rates in HCC patients who were treated by TAE/TACE, and the SMI and PMI can be adopted as predictive indicators. Nonetheless, conclusions varied since other studies reached contradictory opinions on the predictive values of sarcopenia in the prognosis of HCC [7–16]. In addition to the loss of muscle, fatty infiltration of muscle is another important clinical outcome of HCC and is usually related to a worse prognosis, however, evidence from the relative studies were limited and the ultimate conclusion still needs to be further validated [17, 18]. Myosteatosis is used to describe fatty infiltration and can be diagnosed by CT scanning using muscle radiation attenuation.

To further clarify the predictive values of the changes of muscle, both the changes of quantity and quality of muscle in the HCC patients who were treated by TAE or TACE, we conducted a meta-analysis to provide the evidence-based results for the predictive values of sarcopenia, SMI, PMI and the myosteatosis in regard to the impact of survival outcomes and tumor response in HCC patients treated by TAE or TACE, thereby assisting in optimizing therapeutic strategies and maximizing clinical benefits for HCC patients.

## METHODS

### Literature search strategies

The present study was conducted strictly with the instruction of the Preferred Reporting Items for Systematic Reviews and Meta-Analyses (PRISMA) guidelines. In this meta-analysis, databases including PubMed (https://pubmed.ncbi.nlm.nih.gov/), EMBASE (https://www.embase.com/), Cochrane Library (https://www.cochranelibrary.com/) were adopted to complete the literature review before June 19, 2023. Mesh terms and entry terms included “sarcopenia” (Mesh), “skeletal muscle index”, “psoas muscle index”, “SMI”, “PMI”, “subcutaneous adipose index”, “SAI”, “subcutaneous fat index”, “SFI”, “visceral adipose index”, “VAI”, “visceral fat index”, “VFI”, “intramuscular adipose index”, “IMAI”, “intramuscular fat index”, “IMFI”, “muscle surface area”, “MSA”, “skeletal muscle density”, “SMD”, “myosteatosis”, “sarcopenic”, “myopenia”, “transarterial embolization”, “TAE”, “transcatheter intra-arterial therapy”, “transcatheter intra-arterial therapies”, “Transcatheter arterial chemoembolization”, “transarterial chemoembolization”, “trans-arterial chemoembolization”, “TACE”, and those terms were searched. Besides, Google Scholar was also used to complete a gray literature search and we also manually retrieved the relative reference of the qualified publications to find more targeted reports. The specific search strategy in PubMed is shown in Supplementary File 1.

### Inclusion and exclusion criteria

For the literature review, the inclusion criteria include: (1) patients diagnosed with HCC; (2) patients treated with TAE or TACE; (3) studies evaluated the predictive values of sarcopenia, myosteatosis, SMI, and PMI; (4) researches provided at least one of the following outcomes (overall survival (OS), objective response rate (ORR), disease control rates (DCR)). The exclusion criteria include: (1) duplicated reports; (2) conference abstracts, case reports, and comments; (3) studies with overlapping patient populations. Only articles with the most complete data and rigorous methodology were selected.

### Data extraction and quality assessment

Data extraction mainly focused on the authors, study design, study period, treatment, study region, sample size, gender, the age of patients, outcomes, and so on. We used the Newcastle–Ottawa Scale (NOS) score to assess the quality of collected studies [19]. Studies with scores higher than six were classified as high-quality research. All of the above steps, i.e., literature search, screening, data extraction, and literature quality assessment were done independently and cross-checked by two authors, and the senior author was consulted in case of disputes.

### Statistical analysis

Stata 15.0 was used to conduct statistical analyses. The relationships between the sarcopenia, myosteatosis, SMI, PMI and the overall survival in TAE or TACE treated HCC patients were calculated by the hazard ratio (HR) with a 95% confidence interval (95% CI). The odds ratio (OR) with a 95% CI was used to describe the relationship between above indicators and the ORR or DCR in TAE or TACE treated HCC patients. Include four studies reported ORR by combining partial response and complete response. The statistical heterogeneity was calculated using the chi-squared test. *p* < 0.1 and I^2^ > 50% were defined as high heterogeneity and a random effect model would be applied when it occurred. Otherwise, the fixed effect model was used [20]. The tests of Egger [21] and Begg [22] were employed to evaluate publication bias. The sensitivity analysis was conducted by the leave-one-out method to evaluate the stability of results [23].

## RESULTS

### Characteristics of included studies

**Figure 1:**
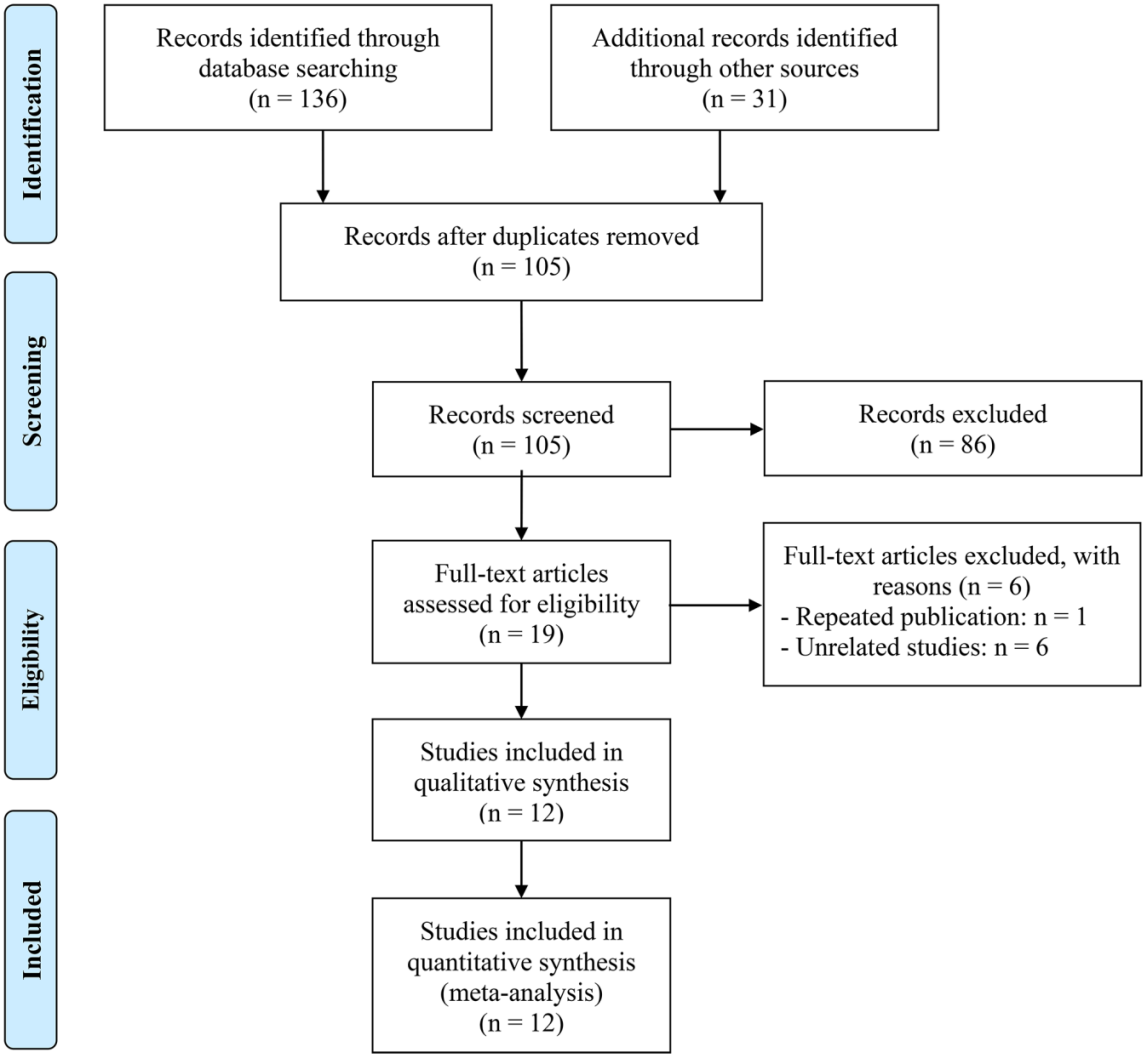
The flowchart of the literature review.

As shown in Figure 1, a total of 167 studies were collected. After removing 62 duplicate articles, 105 studies were assessed by title and abstract with 19 qualified articles. After reading full texts, one repeated publication and 6 unrelated studies were excluded. 12 studies involving 2559 participants were included in the systematic review ultimately. More detailed information about the eligible assessment of studies is provided in Figure 1. The baseline information of included studies is shown in Table 1. All the studies were evaluated with scores of more than six, which represented high-quality studies. Included in all studies was the use of CT to assess body composition at the third lumbar level.

**Table 1:** Main characteristics of the studies included.

**Study**	**Study design**	**Study period**	**Treatment**	**Study region**	**Sample size**	**Gender (male/female)**	**Age (years)**	**Outcomes**	**Methods, sites, and cut-off (male/female)**	**NOS Score**
Wang et al. 2023	R	06/2013–12/2019	TACE	China	364	300/64	58 (50–66)^a^	SMI (OS)	CT, L3, 49/36 cm^2^/m^2^	7
Loosen et al. 2023	R	2011–2021	TACE	Germany	89	61/28	69 (23–90)^b^	SMI (OS)	CT, L3, CV	6
Bannangkoon et al. 2023	R	01/2008–12/2019	TACE	Thailand	611	445/166	61.4 ± 10.9	SMI (OS, ORR), Myosteatosis (OS)	CT, L3, 36.2/29.6 cm^2^/m^2^; 44.4/39.3 HU	7
Zhang et al. 2022	R	01/2018–03/2019	TACE	China	228	175/53	58.9 ± 11.0	SMI (OS), PMI (OS)	CT, L3, 46/34 cm^2^/m^2^; 42.3/37.4 mm/m2	8
Yang et al. 2022	R	01/2015–12/2020	TACE	China	62	49/13	59.4 ± 10.6	SMI (OS)	CT, L3, 42/38 cm^2^/m^2^	6
Roth et al. 2022	R	12/2007–12/2014	TACE/TAE	France	225	200/25	65 (58–75)^a^	SMI (OS, ORR, DCR)	CT, L3, 50/39 cm^2^/m^2^	7
Masetti et al. 2022	R	03/2011–07/2019	TAE	Italy	151	116/35	73.2 ± 9.3	Myosteatosis (OS)	CT, L3, 0.44/0.31^c^	7
Chien et al. 2022	R	01/2010–08/2015	TACE	Taiwan	260	192/68	64.0 ± 18.0	PMI (OS)	CT, L3, 6.4/3.9 cm^2^/m^2^	8
Li et al. 2021	R	2008–2018	TACE	China	192	157/35	60 (52–67)^a^	SMI (OS)	CT, L3, CV	7
Lanza et al. 2020	R	03/2011–07/2019	TAE	Italy	142	110/32	73 (40–88)^b^	SMI (OS)	CT, L3, 55/39 cm^2^/m^2^	7
Loosen et al. 2019	R	2013–2018	TACE	Germany	56	44/12	65 (30–89)^b^	PMI (OS, ORR)	CT, L3, 11.8/11.8 mm/m^2^	6
Fujita et al. 2019	R	01/2006–03/2017	TACE	Japan	179	130/49	72 (64–78)^a^	PMI (OS, ORR, DCR)	CT, L3, 6.0/3.4 cm^2^/m^2^	7

^a^medians (interquartile range); ^b^medians (ranges); ^c^myosteatosis were intramuscular adipose tissue content (IMAC) > −0.44 in males and > −0.31 in females. Abbreviations: R: retrospective analysis; TACE: trans-arterial chemoembolization; TAE: trans-arterial embolization; SMI: skeletal muscle index; PMI: psoas muscle index; OS: overall survival; ORR: objective response rate; DCR: disease control rates; L3: third lumbar vertebra; CT: computed tomography; CV: continuous variable.

### The predictive value of sarcopenia in TAE or TACE treated HCC patients

**Figure 2:**
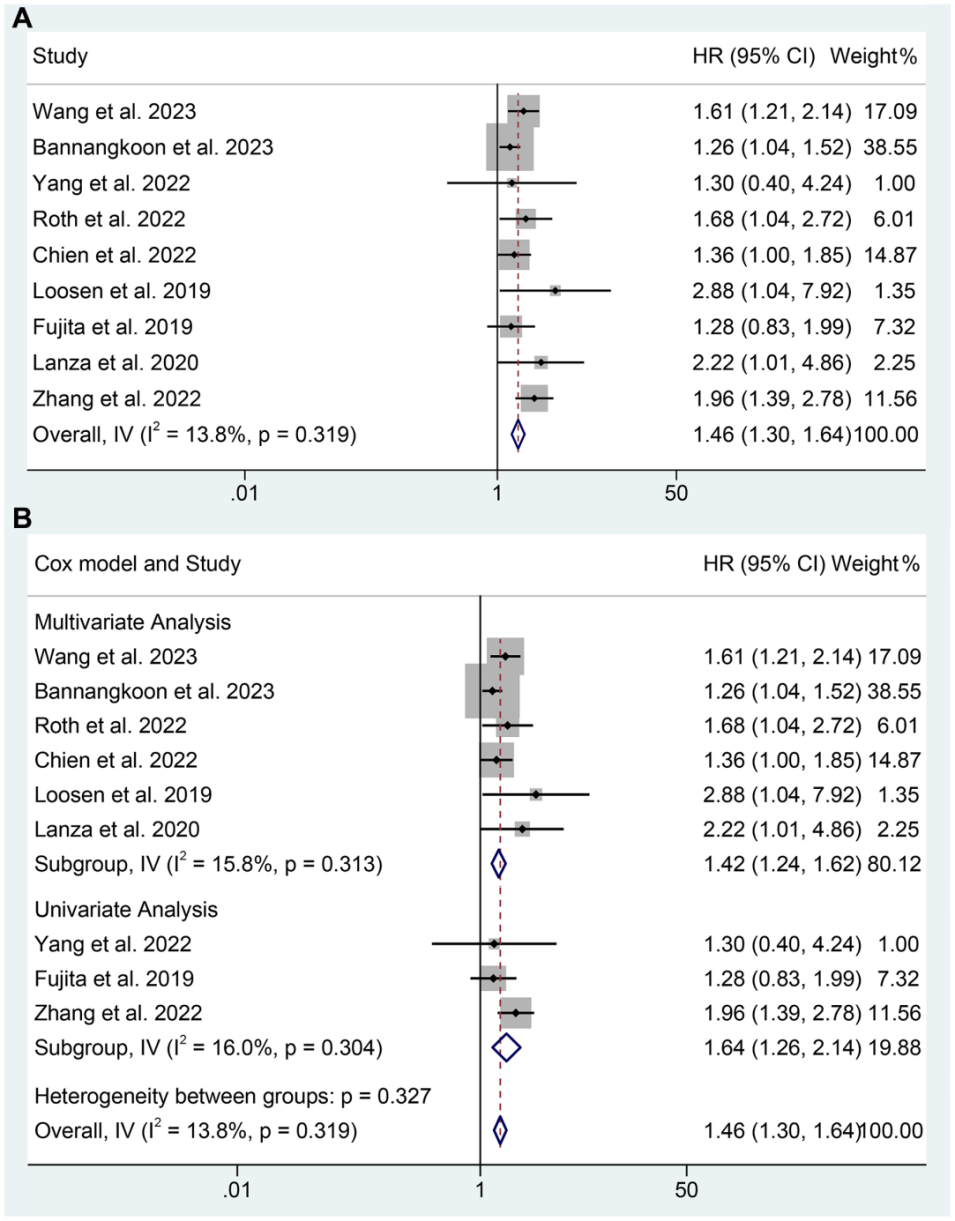
**The relationship between sarcopenia and OS in TAE or TACE treated HCC patients.** (**A**) The overall analysis; (**B**) Subgroup analysis including multivariate analysis and univariate analysis.

Eight studies including 2559 patients reported the relationship between sarcopenia and overall survival. There was no significant heterogeneity found among included studies, so a fixed-effect model was applied. As shown in Figure 2A, the meta-analysis assisted that the sarcopenia was significantly related to a shorter OS of HCC patients who were treated by TAE or TACE (HR: 1.46, 95% CI: 1.30–1.64, *p* < 0.001). In the subgroup analysis, six studies using multivariate analysis supported that the sarcopenia was devoted to a shorter OS (HR: 1.42, 95% CI: 1.24–1.62, *p* < 0.001). In the univariate analysis group, which included only three studies, sarcopenia was also significantly associated with shorter OS (HR: 1.64, 95% CI: 1.26–2.14, *p* < 0.001). In all, sarcopenia could predict a shorter OS in HCC patients who were treated by TAE or TACE. More detailed information about the subgroup analysis is provided in Figure 2B.

**Figure 3:**
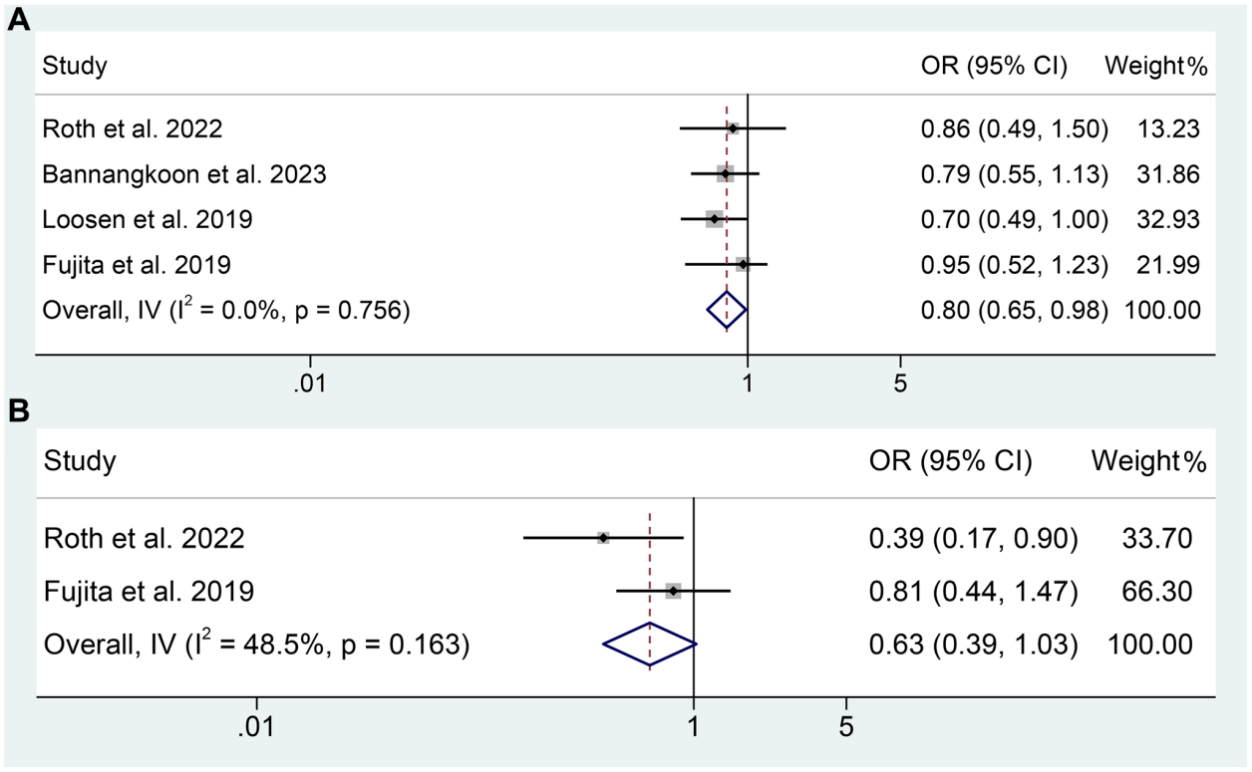
(**A**) The relationship between sarcopenia and ORR in TAE or TACE treated HCC patients; (**B**) The relationship between sarcopenia and DCR in TAE or TACE treated HCC patients.

Apart from the OS, four studies provided the ORR and 2 studies provided DCR of TAE or TACE treated HCC patients. As shown in Figure 3, the meta-analysis suggested that sarcopenia was significantly associated with lower ORR of TAE or TACE treated HCC patients (OR: 0.80, 95% CI: 0.65–0.98, *p* = 0.032). However, there was no statistical difference in DCR (OR: 0.63, 95% CI: 0.39–1.03, *p* = 0.064). In consideration of the limited sample size and studies of DCR, the conclusion should be further validated.

The Cochran *Q* test and I^2^ statistics showed no significant heterogeneity (overall OS: I^2^ = 13.8%, *p* = 0.319; multivariate analysis of OS: I^2^ = 15.8%, *p* = 0.313; univariate analysis of OS: I^2^ = 16.0%, *p* = 0.304; ORR: I^2^ = 0, *p* = 0.756; DCR: I^2^ = 48.5%, *p* = 0.163), thus a fixed-effect model was utilized.

### The predictive value of SMI in TAE or TACE treated HCC patients

**Figure 4:**
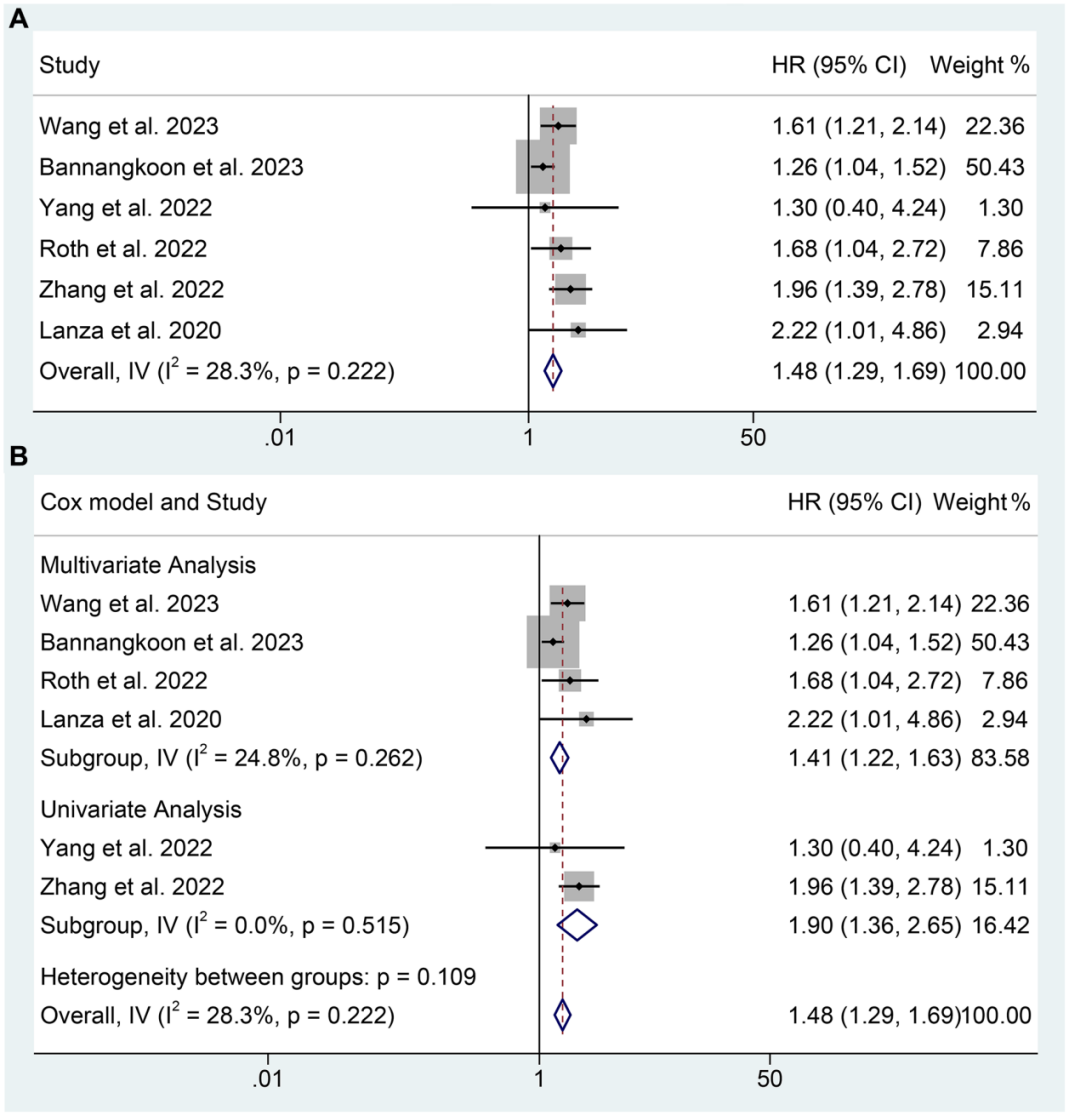
**The relationship between SMI and OS in TAE or TACE treated HCC patients.** (**A**) Taking the SMI as a binary variable; (**B**) Subgroup analysis of figure A.

In the meta-analysis, six studies provided the SMI level of patients. Taking the SMI as a binary variable, the low SMI was related to a shorter OS of TAE or TACE treated HCC patients (HR: 1.48, 95% CI: 1.29–1.69, *p* < 0.001). The subgroup analysis reached the same conclusion (multivariate analysis, HR: 1.41, 95% CI: 1.22–1.63, *p* < 0.001; univariate analysis, HR: 1.90, 95% CI: 1.36–2.65, *p* < 0.001). In conclusion, the SMI could effectively predict the OS of TAE or TACE treated HCC patients (Figure 4). There was no significant heterogeneity was found (overall OS: I^2^ = 28.3%, *p* = 0.222; multivariate analysis of OS: I^2^ = 24.8%, *p* = 0.262; univariate analysis of OS: I^2^ = 0, *p* = 0.515), thus a fixed-effect model was used.

Besides, three studies by Loosen et al. (HR: 0.90, 95% CI: 0.83–0.98, *p* = 0.014), Roth et al. (HR: 0.99, 95% CI: 0.97–1.01, *p* = 0.19), and Li et al. (HR: 0.98, 95% CI: 0.97–1.00, *p* = 0.028), which considered SMI as a continuous variable, also found the lower SMI contributed to the shorter OS of TAE or TACE-treated HCC patients.

### The predictive value of PMI and myosteatosis in TAE or TACE treated HCC patients

**Figure 5:**
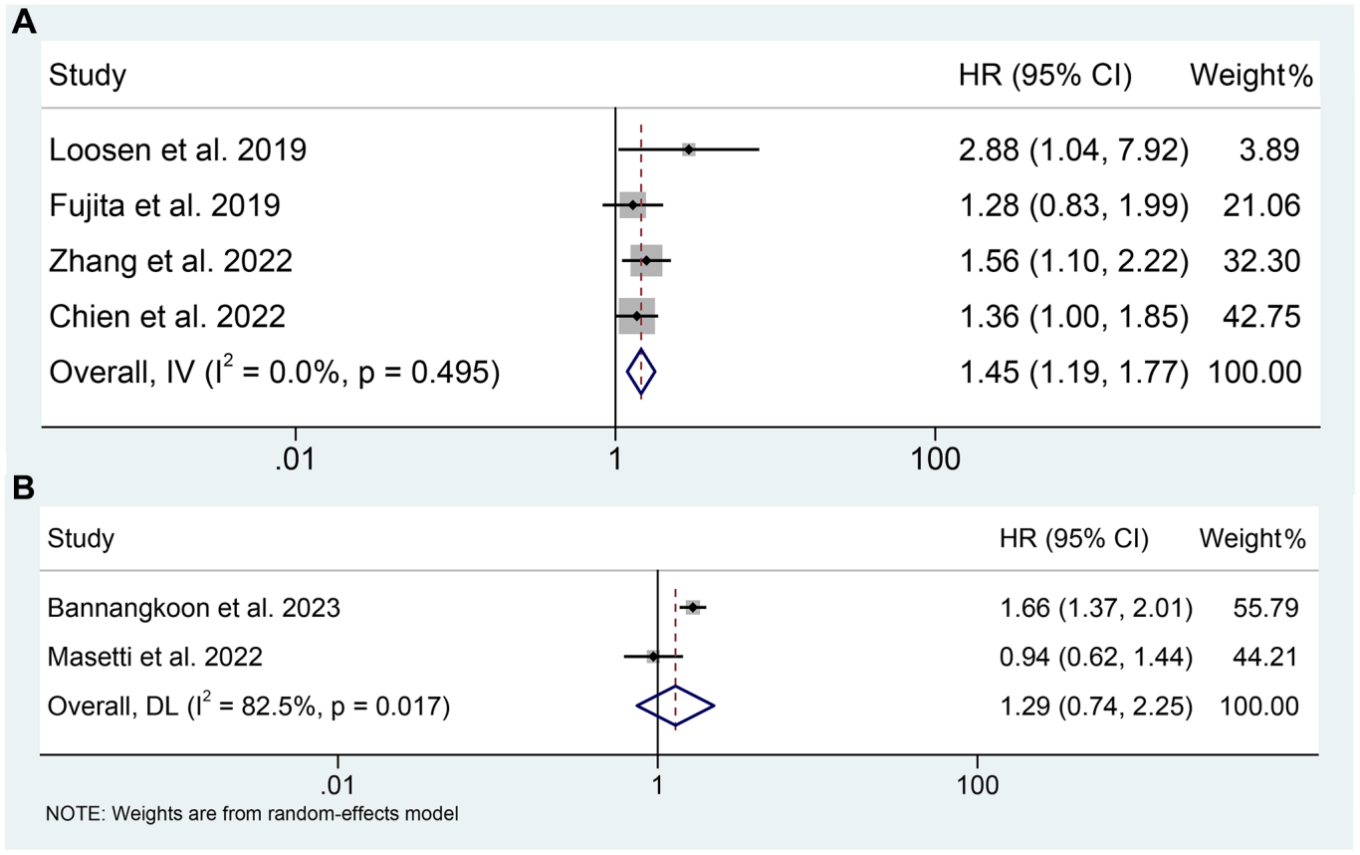
(**A**) The relationship between PMI and OS in TAE or TACE treated HCC patients; (**B**) The relationship between myosteatosis and OS in TAE or TACE treated HCC patients.

As for the PMI, four studies reported the relationship between the PMI and OS of TAE or TACE treated HCC patients. As shown in Figure 5A, the meta-analysis results indicated that lower PMI was closely related to a shorter OS (HR: 1.45, 95% CI: 1.19–1.77, *p* < 0.001). No significant heterogeneity was observed among the included studies (I^2^ = 5.8%, *p* = 0.495). As a result, a fixed-effects model was employed.

After the literature review, there were two studies reported the relationship between the myosteatosis and the OS of TAE or TACE treated HCC patients. However, their conclusions were contradictory. The meta-analysis using pooled data from 762 patients supported that there was no significant association between myosteatosis and OS (HR: 1.29, 95% CI: 0.74–2.25, *p* = 0.366). More detailed information is provided in Figure 5B. There was an obvious heterogeneity among the included studies and a random-forest model was adopted for analysis.

### Publication bias and sensitivity analysis

**Figure 6:**
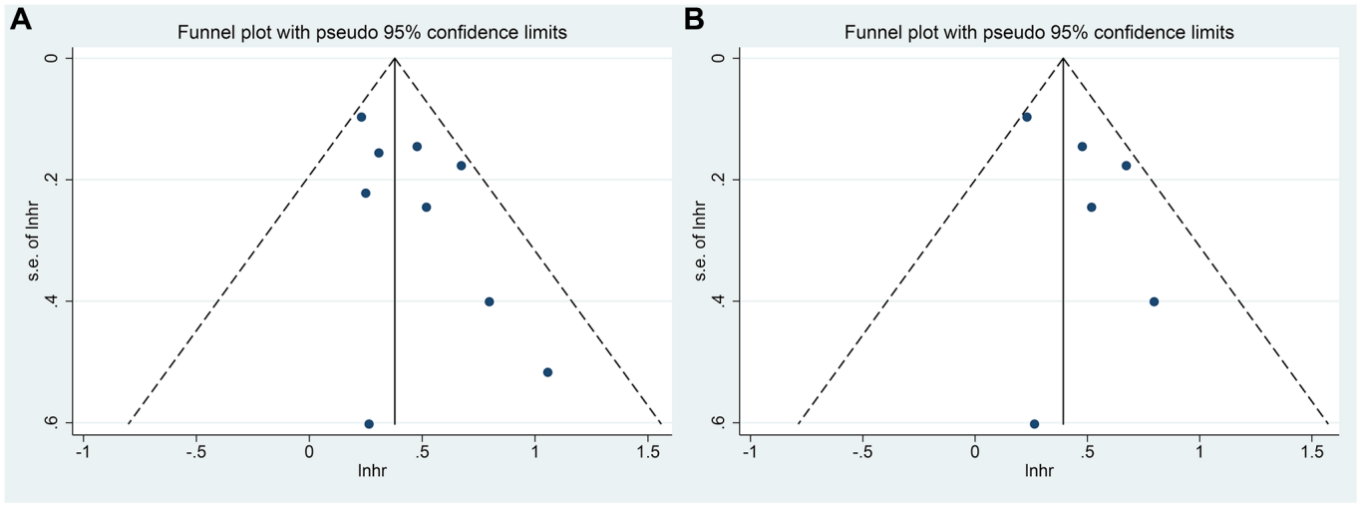
(**A**) The funnel plot of the impact of sarcopenia on OS in TAE or TACE treated HCC patients; (**B**) The funnel plot of the impact of SMI on OS in TAE or TACE treated HCC patients.

The funnel plot was provided in Figure 6 which described the publication bias. There was no significant publication bias found in Egger’s test (sarcopenia and OS, *p* = 0.109; SMI and OS, *p* = 0.234) or Begg’s test (sarcopenia and OS, *p* = 0.348; SMI and OS, *p* = 1.000).

**Figure 7:**
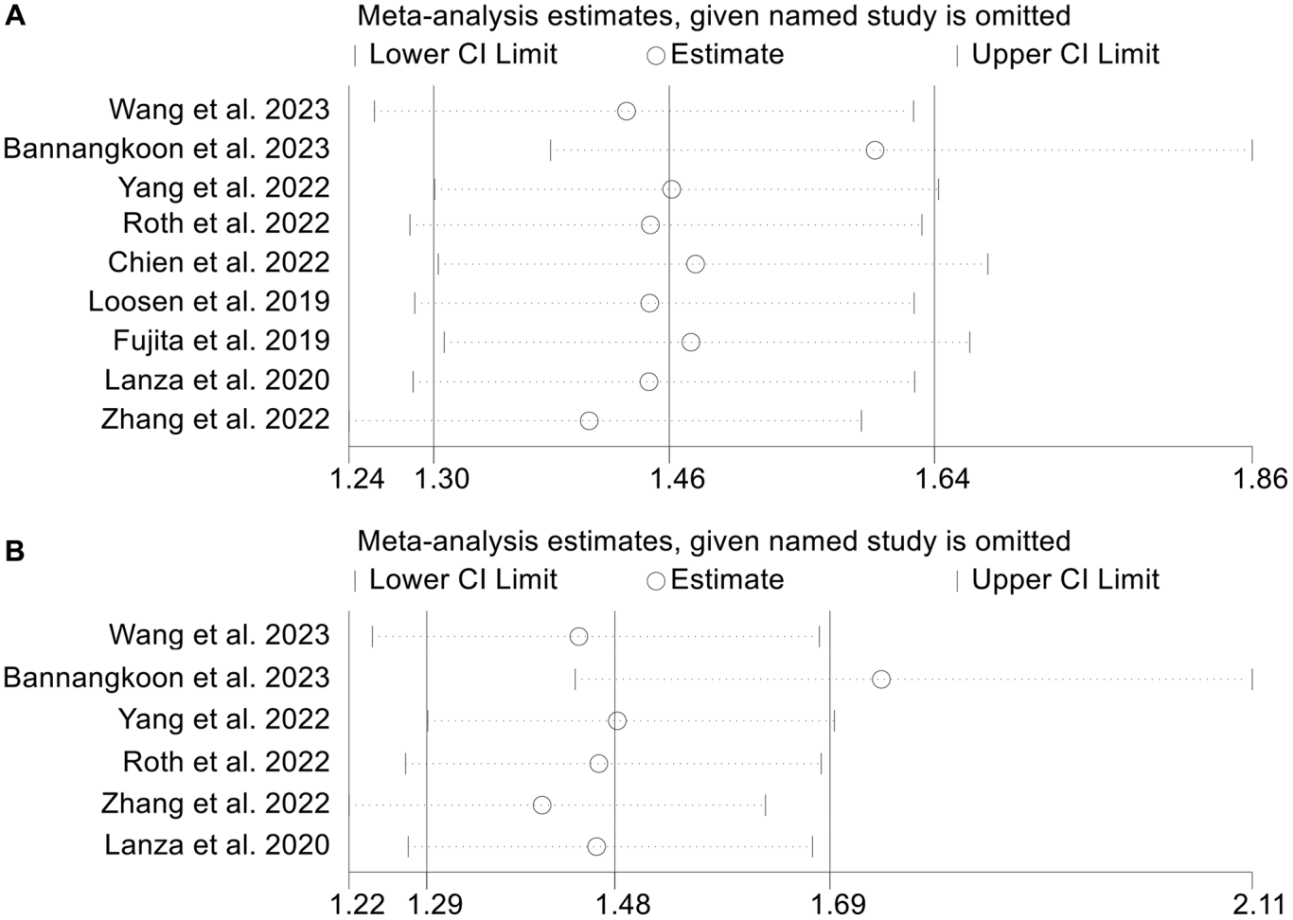
(**A**) The sensitivity analysis of the impact of sarcopenia on OS in TAE or TACE treated HCC patients; (**B**) The sensitivity analysis of the impact of SMI on OS in TAE or TACE treated HCC patients.

In the present study, we adopted the leave-one-out method to conduct the sensitivity analysis to evaluate the impact of each included study on the combined results. The impact of sarcopenia and SMI on the OS of TAE/TACE treated HCC patients was the primary endpoint that we investigated in the meta-analysis, as a result, we conducted the sensitivity analysis of studies concerning the relationship between sarcopenia, SMI and the OS. HR from the pooled data has not significantly changed after excluding one study at a time, ranging from 1.40 (95% CI: 1.24–1.59, after omitting Zhang et al. 2022) to 1.60 (95% CI: 1.38–1.86, after omitting Bannangkoon et al. 2023). A similar conclusion was reached in the SMI, of which the HR ranged from 1.41 (95% CI: 1.22–1.63, after omitting Zhang et al. 2022) to 1.75 (95% CI: 1.44–2.11, after omitting Bannangkoon et al. 2023). The aforementioned analysis proved that conclusions of the meta-analysis we conducted were stable and reliable. Figure 7 provides detailed information on the sensitivity analysis.

## DISCUSSION

In the present study, we conducted a meta-analysis with several subgroup analyses to investigate the relationship between sarcopenia, SMI, PMI, myosteatosis, and the prognosis of TAE or TACE treated HCC patients. Sarcopenia is a significant predictive indicator for the worse prognosis of TAE or TACE treated HCC patients both in the OS and the ORR. As important diagnostic indicators of sarcopenia, the SMI and PMI could also effectively predict the OS of TAE/TACE treated HCC patients. Different from sarcopenia which mainly represents the progressive loss of muscle mass and strength, myosteatosis is used to describe the fatty infiltration of muscle assessed by CT scanning, both in myocytes (intramyocellular fat) and in muscle fascia (intermuscular fat). Different from SMI, PMI and sarcopenia, the present meta-analysis didn’t find any impact of myosteatosis on OS of TAE or TACE treated HCC patients. Hence, may it be the loss of muscle rather than the fatty infiltration of muscle that contributes to the poor prognosis of TAE/TACE treated HCC patients. However in consideration of the limited number of myosteatosis studies, the conclusion should be further validated.

Previous studies pointed out that sarcopenia was related to worse outcomes when patients underwent major surgery [5, 24, 25] and it was an unfavorable prognostic factor for patients with cirrhosis and HCC treated by TAE or TACE [7]. It remains debatable whether sarcopenia could impact the prognosis of HCC undergoing TAE or TACE. Some studies did not find a relationship between sarcopenia and prognosis of HCC patients [8, 16]. In contrast, studies with larger sample sizes and lower heterogeneous patient populations revealed that sarcopenia was an independent predictive indicator for TAE/TACE treated HCC patients [7, 10, 12–14, 17]. In our meta-analysis, pooled data from 1932 patients supported that sarcopenia was an independent poor prognostic factor for TAE or TACE treated HCC patients both in OS and in ORR. Usually, we can observe a high prevalence of sarcopenia in TAE or TACE treated HCC patients. Especially, HCC in sarcopenic patients was in a more advanced stage with larger tumor size and number [6]. Thus, along with TACE or not, systemic therapy with either tyrosine kinase inhibitors checkpoint inhibitors, or radiotherapy should be considered for sarcopenic patients with unresectable HCC in an advanced stage to maximize the benefits for patients [26, 27]. It is noteworthy that even though sarcopenia is an independent poor prognostic factor of TAE or TACE treated HCC patients, it is not associated with a higher rate of post-TAE or TACE complications so that it does not impair the safety of the TAE or TACE. Therefore, sarcopenia should not be treated as an exclusion criterion for TAE or TACE in HCC patients [13].

Older age, male tendency, and lower BMI were found to be closely related to sarcopenia. Nasimi et al. found older age and lower BMI increased the risk of SMI. As the main diagnostic indicator of sarcopenia, SMI has cut-off values for sarcopenia at ≤39 cm^2^/m^2^ for women and ≤55 cm^2^/m^2^ for men. When taking SMI as a binary variable following the above cut-off value, it could effectively predict the OS of TAE or TACE treated HCC patients according to present meta-analysis. In clinical practice, more efforts should be put into the increase of SMI to improve the OS of TAE or TACE treated HCC patients. According to previous studies, physical exercises could help to strengthen the muscles and significantly increase SMI [28, 29]. Similar to SMI, PMI, as one of the important indicators for muscle loss and the other diagnostic criterion of sarcopenia, also demonstrated a close association with prognosis in HCC in the study. Both SMI and PMI are famous prognostic predictors and are simple to assess. However, in previous studies, SMI seemed to be more robust and it could completely measure the muscle mass and was more popular in predicting the prognosis of various diseases compared to PMI [11, 13, 30].

Myosteatosis was also highly prevalent in HCC patients and has been proven to be an important prognostic factor for hepatobiliary and pancreatic malignancies [31]. However, its impact on prognosis is straightforward. In most relevant studies, myosteatosis did not demonstrate any impact on OS and complications [31]. In our study, the included two studies reached contrary conclusions. Bannangkoon et al. found myosteatosis was associated with the OS of TAE or TACE treated HCC patients [17], but the study from Masetti et al. pointed out there was no significant impact of myosteasis on the OS of TAE or TACE treated HCC patients [18], which was consistent with our meta-analysis. In consideration of the limited sample size and the long period of Masetti’s study, the impact of myosteasis on OS in TAE or TACE treated HCC should be further validated with a multicenter, larger sample size study in different populations.

There are some inherent limitations in our study. Firstly, the meta-analysis was conducted on published studies and the limited sample size constrained us from detailed subgroup analysis. There were only two studies involving myosteasis and the OS in TAE or TACE treated HCC. Also, the analysis of SMI, PMI, and myosteasis on ORR, and DCR was absent due to the lack of relevant studies. Secondly, all included studies were retrospectively designed. More strict prospective trials should be required to exclude confounding influence. Lastly, included studies of SMI, PMI and myosteasis did not adopt the same cut-off value for diagnosis, which should be optimized.

In conclusion, the present meta-analysis proved that sarcopenia with its diagnosis indicators, SMI and PMI were significant prognostic factors for TAE or TACE treated HCC patients. While myosteasis has a non-prognostic impact on the OS of TAE or TACE treated HCC patients. The findings could support more comprehensive therapeutic strategies to maximize the benefits for HCC patients in clinical practice.

## Supplementary Materials

Supplementary File 1
