# Supplementary material for: The predictive value of sarcopenia and myosteatosis in trans-arterial (chemo)-embolization treated HCC patients
Source: Aging (Albany NY). 2024 Jan 5;16(1):389–401. doi: 10.18632/aging.205375 (PMC10817392; doi:10.18632/aging.205375)
Supplement: Supplementary File 1 [file aging-16-205375-s001.pdf]

SUPPLEMENTARY MATERIALS

Supplementary File 1. Search strategies in PubMed.

(((((((((transarterial embolization[Title/Abstract]) OR (TAE[Title/Abstract])) OR (transcatheter intra-arterial therapy[Title/Abstract])) OR (transcatheter intra-arterial therapies[Title/Abstract])) OR (Transcatheter arterial chemoembolization[Title/Abstract])) OR (transarterial chemoembolization[Title/Abstract])) OR (trans-arterial chemoembolization[Title/Abstract])) OR (TACE[Title/Abstract]))) AND (((sarcopenias) OR (((((((((((((((((((((((((skeletal muscle index) OR (SMI)) OR (psoas muscle index)) OR (PMI)) OR (subcutaneous adipose index)) OR (SAI)) OR (subcutaneous fat index)) OR (SFI)) OR (visceral adipose index)) OR (VAI)) OR (visceral fat index)) OR (VFI)) OR (intramuscular adipose index)) OR (IMAI)) OR (intramuscular fat index)) OR (IMFI)) OR (muscle surface area)) OR (MSA)) OR (skeletal muscle density)) OR (SMD)) OR (myosteatosis)) OR (sarcopenia)) OR (sarcopenic)) OR (Myopenia)))) OR (“Sarcopenia”[Mesh]))
